# 粪菌移植：肺癌治疗的潜在途径

**DOI:** 10.3779/j.issn.1009-3419.2025.101.21

**Published:** 2025-11-20

**Authors:** HONG Xiuwen, DENG Yaqian, FENG Jiao, BAO Cui, ZHANG Yuanyuan, GAO Nan, SHEN Hong

**Affiliations:** 210003 南京，南京医科大学第二附属医院呼吸与危重症医学科; Department of Respiratory and Critical Care Medicine, The Second Affiliated Hospital of Nanjing Medical University,Nanjing 210003, China

**Keywords:** 肺肿瘤, 粪菌移植, 肠道微生物, Lung neoplasms, Fecal microbiota transplantation, Gut microbiota

## Abstract

随着肺癌治疗的不断发展，手术、放化疗、新辅助治疗、靶向治疗及免疫治疗等主流手段在临床中被广泛应用，但仍存在疗效局限性和明显副作用。近年来，肠道菌群在肿瘤免疫调控中的关键作用日益凸显，其对肿瘤免疫治疗的潜在影响已成为肺癌治疗的新兴研究热点。在此背景下，粪菌移植（fecal microbiota transplantation, FMT）作为一种潜在的免疫调节策略被提出，它通过调控肠道菌群以增强宿主免疫应答，改善肿瘤免疫微环境。本文系统梳理了FMT治疗肺癌的最新研究进展，主要围绕肠道菌群与肺癌的关联性、FMT治疗作用机制及临床应用研究等方面，全面探讨了FMT在肺癌治疗中面临的挑战与应用前景。

## Abstract (en)

As lung cancer treatment continues to advance, mainstream approaches such as surgery, radiotherapy, chemotherapy, neoadjuvant therapy, targeted therapy and immunotherapy have become widely adopted in clinical practice. However, the efficacy of these methods is still limited and they are associated with significant side effects. In recent years, the pivotal role of the gut microbiota in tumor immune regulation has become increasingly recognized, with its potential impact on tumor immunotherapy emerging as a novel therapeutic focus in lung cancer management. Against this backdrop, fecal microbiota transplantation (FMT) has been proposed as a potential immunomodulatory strategy. It enhances host immune responses and improves the tumor immune microenvironment by regulating the gut microbiota. This paper provides a systematic review of the latest research advances in FMT for lung cancer treatment. Focusing on the relationship between gut microbiota and lung cancer, the therapeutic mechanisms of FMT and clinical application studies, it provides a comprehensive exploration of the challenges and prospects for the use of FMT in lung cancer therapy.

肺癌是全球发病率和死亡率最高的常见恶性肿瘤。据国际癌症研究机构（International Agency for Research on Cancer, IARC）统计^[1]^，2022年全球范围内肺癌新发人数约达248万。同年中国新发肺癌病例约达106.06万例，数量居世界首位，且肺癌发病率在国内所有恶性肿瘤中位列第一^[2]^。目前，仅约30%的肺癌患者在初诊时处于早期阶段，多数患者确诊时已进展至晚期^[3]^。晚期肺癌的治疗除传统的化疗、放疗外，免疫检查点抑制剂（immune checkpoint inhibitors, ICIs）、抗血管生成治疗等靶向治疗新手段明显延长了患者生存期^[4]^。此外，新型的抗体偶联药物（antibody-drug conjugate, ADC）、双特异性抗体给晚期肺癌带来新的治疗选择和获益^[5]^。尽管如此，患者仍不可避免地面临复发或疾病进展的挑战，且药物的不良反应常影响患者治疗的持续性甚至危及生命^[6]^。近年的资料^[7]^显示，肺癌总体5年生存率仍处于较低水平（生存率26.4%），距国家《健康中国2030规划纲要》提出的46.6%的目标较为遥远。由此凸显了开发创新性治疗策略的迫切需求。

粪菌移植（fecal microbiota transplantation, FMT）通过将健康供者粪便微生物转移至患者体内，旨在重建肠道菌群稳态以优化临床疗效^[8]^。其实，现代科学利用粪便治病并非首创。早在明代，李时珍《本草纲目》中记载“粪清”可用于治疗中毒以及消化道疾病。尽管古代理论体系与现代技术截然不同，但这无疑是人类对于微生物疗法的一次大胆创新和探索^[9]^。数世纪后现代医学基于对微生物学的深刻理解，发展出“FMT”。近年来，FMT在肺癌治疗领域展现出潜在的应用价值^[10]^。本文全面评估了FMT作为一种前沿治疗模式的作用机制及其在肺癌临床实践中的转化应用挑战。

## 1 肠道菌群及其肠-肺轴相互作用机制

人体结肠内栖息着约100万亿个、密度高达每毫升10¹¹至10¹²个细胞的复杂微生物群落，构成一个营养丰富的动态微生态系统^[11]^。胃肠道和肺部虽然解剖位置相异，但均起源于胚胎前肠内胚层，并共享高度相似的黏膜组织结构，这为二者形成肠-肺轴这一双向通信奠定了基础^[12]^。肠道菌群以其高度多样性和共生菌与致病菌（如梭状芽胞杆菌、埃希菌属等）的精细平衡，共同维持内环境稳态^[13]^。其中，益生菌（如双歧杆菌、乳酸杆菌等）作为一类对人体具有多重益处的有益微生物，可通过产生细菌素、乳酸和过氧化氢等抗菌化合物抑制病原体，并发挥广泛的免疫调节、抗癌与代谢功能^[14]^。

肠道与肺部微生物群通过淋巴系统和循环系统构建跨器官的微生物调控网络。当肺泡-毛细血管屏障完整性受损后，肠源性机会病原体（如肠杆菌科）及其DNA片段可易位至体循环，从而扰乱菌群稳态^[15,16]^。短链脂肪酸（short-chain fatty acids, SCFAs）作为肠道菌群的关键代谢产物，是介导肠-肺轴的核心信使分子，它们通过血液和淋巴系统触发远程免疫对话。一方面，刺激肠上皮细胞促进免疫因子释放，招募黏膜免疫细胞向肺组织迁移^[17]^；另一方面，以丁酸盐为代表的SCFAs通过诱导细胞凋亡维持肠道屏障完整性^[18]^，增加CD8^+^细胞毒性T淋巴细胞抗肿瘤活性，并通过提升Tbx21/干扰素-γ（interferon-γ, IFN-γ）启动子组蛋白H4乙酰化水平、抑制组蛋白去乙酰化酶活性，促进IFN-γ分泌^[19]^。此外，SCFAs还能通过抑制组蛋白去乙酰化酶激活哺乳动物雷帕霉素靶蛋白（mammalian target of rapamycin, mTOR）通路，调控T细胞分化^[20]^；丁酸盐则通过增加Foxp3乙酰化以稳定其表达，有助于维持免疫耐受^[21]^。在肺部微环境中，SCFAs与细菌脂多糖（lipopolysaccharide, LPS）可上调肺泡巨噬细胞G蛋白偶联受体表达^[17]^，同时SCFAs抑制核因子-κB（nuclear factor-κB, NF-κB）信号通路，驱动巨噬细胞向M2抗炎表型极化，减少白细胞介素（interleukin, IL）-6/肿瘤坏死因子-α（tumor necrosis factor-α, TNF-α）释放并促进IL-10分泌^[22]^。研究^[23]^还发现，丁酸盐对肺癌细胞增殖具浓度依赖性双向调控：低浓度（1 mmol/L）促增殖，高浓度（5 mmol/L）抑增殖。此外，SCFAs缺乏会导致肠道通透性增加，促使细菌产物（如LPS）系统性易位，可能破坏代谢通路而参与癌症的起始与进展^[24]^，这些发现标志着该领域正从相关性向因果性深化。

肠-肺轴核心机制在于肠道菌群对肺部免疫的系统性调控作用。肠道免疫细胞捕获细菌后，抗原呈递细胞则将其运输至肠系膜淋巴结，激活B/T细胞并触发炎症小体活化、树突状细胞迁移及T细胞分化的级联反应，最终引发系统性免疫应答的重编程^[25]^。此外，肠道菌群能精细调控Treg细胞，影响抗肿瘤免疫。易位的菌群成分激活Toll样受体（Toll-like receptor, TLR）信号，不仅增强T细胞增殖与肿瘤特异性记忆应答，且肿瘤微环境中基质/肿瘤细胞分泌的趋化因子（如CCL20/CCL28）可通过CCR6/CCR10受体介导Tregs向肿瘤部位的定向募集，助力形成免疫抑制微环境^[26]^。

## 2 肠道菌群在肺癌发生发展中的潜在作用机制

肠-肺轴是一个复杂的互作网络，肠道菌群失调可通过多途径参与肺癌的发生与发展，包括对关键致癌信号通路的干预、肿瘤相关炎症微环境的塑造以及直接诱导宿主细胞DNA损伤等核心生物学过程（图1）。

**图 1:**
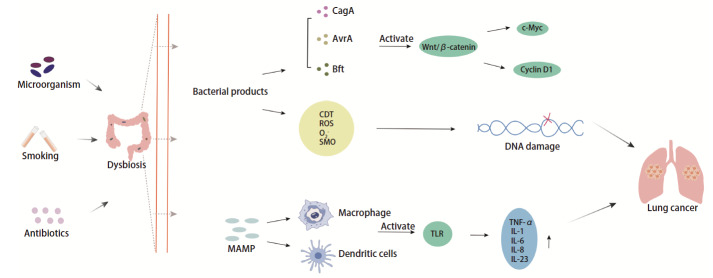
肠道微生物群定植及肺部肿瘤形成

Wnt/β-catenin等致癌性信号通路促进肿瘤细胞存活与增殖，其异常激活是肺癌发生发展的关键驱动力之一^[27]^。β-catenin参与肿瘤调控，肠道菌群扰动可释放其致癌潜能。多种肠道微生物已被证实可激活该通路。幽门螺杆菌分泌的细胞毒素相关基因A（cytotoxin-associated gene A, CagA）和伤寒沙门氏菌分泌的沙门氏菌抗毒力因子A（avirulence A, AvrA）以及由脆弱拟杆菌分泌的B. fragilis毒素（Bacteroides fragilis toxin, BFT）可通过介导E-cadherin裂解或者直接促使β-catenin信号转导^[28-31]^，进而上调c-Myc、Cyclin D1等下游靶基因，驱动细胞周期进程和肿瘤增殖^[32]^。

特定微生物-炎症信号轴通过维持慢性促炎微环境加速肺癌进展。当肠道屏障损伤引起细菌易位时，其微生物相关分子模式（microbe-associated molecular patterns, MAMPs）可激活巨噬细胞和树突状细胞中的TLR^[33]^。TLRs在识别病原体相关分子模式（pathogen-associated molecular patterns, PAMPs）和损伤相关分子模式（damage-associated molecular patterns, DAMPs）后，触发MYD88、丝裂原活化蛋白激酶（mitogen-activated protein kinases, MAPK）等信号分子激活NF-κB、AP-1等核心信号通路，并释放TNF-α、IL-6及血管内皮生长因子（vascular endothelial growth factor, VEGF），它们不仅直接维系肿瘤细胞存活，更通过持续血管生成塑造促肿瘤炎症微环境^[34,35]^。除此之外，TLR信号可交叉激活肺癌中高频突变的磷脂酰肌醇3-激酶/蛋白激酶B（phosphatidylinositol 3-kinase/protein kinase B, PI3K/AKT）促生存通路，形成恶性进展的正反馈环路^[36]^。

DNA损伤亦是肿瘤发生的另一重要机制。肠道菌群可通过基因毒性作用直接驱动宿主基因组的不稳定性^[37]^：携带pks^+^的大肠杆菌可诱导基因突变^[38]^；表达UshA基因毒素的大肠杆菌可引起DNA双链断裂^[39]^；空肠弯曲菌产生的细胞致死性膨胀毒素（cytolethal distending toxin, CDT）促进β-catenin核转位引起DNA损伤^[40]^；脆弱拟杆菌分泌的BFT上调精胺氧化酶（spermine oxidase, SMO），导致DNA氧化损伤^[41]^；粪肠球菌产生细胞外超氧化物O_2_^-^和活性氧（reactive oxygen species, ROS）通过破坏上皮细胞完整性，引起DNA断裂与突变积累^[42]^。这些机制都能显著提升肺癌发生风险。

## 3 肺癌患者肠道菌群的改变

肠道菌群构成一个动态生态系统，其结构与功能受到包括吸烟与肺癌在内的多种病理生理因素影响。吸烟是肺癌的主要致病因素，不仅通过气道致癌物（如尼古丁）直接致瘤，也被观察到与肠道菌群α-多样性下降等稳态失衡有关联^[43]^。在肺癌进展中，患者肠道菌群呈现特征性失调模式。Zhang等^[44]^发现肺癌患者粪便细菌中厚壁菌门和变形菌门的丰度降低，而拟杆菌门和梭菌门的丰度相对较高。Sun等^[45]^发现在广泛期小细胞肺癌（extensive-stage small cell lung cancer, ES-SCLC）患者中厚壁菌门和拟杆菌门较少。经免疫治疗后，厚壁菌门在门水平上富集，R组有13种属级细菌富集，如普拉梭菌、Subdoligranulum、Clostridium_sensu_stricto_1等。这种失衡在疾病晚期及恶病质癌症中尤为突出。与健康对照组相比，尽管α-多样性未发生显著变化，但β-多样性呈现差异，且韦荣球菌属与未分类的肠杆菌科菌属的丰度与体重减轻程度呈正相关；巨单胞菌属和消化球菌属则在非恶病质患者群体中富集^[46]^。这些微生物组变化与病理类型、疾病严重程度紧密耦合，为其作为分层诊疗生物标志物及菌群靶向治疗提供了理论依据。

## 4 基于肠道菌群调控的肺癌治疗

### 4.1 肠道菌群调控策略：从益生菌到FMT的演进

近年来，益生菌已成为癌症治疗中一种潜在的辅助策略，但仍存在争议。益生菌暴露未显著影响ES-SCLC患者的中位无进展生存期（median progression-free survival, mPFS）或中位总生存期（median overall survival, mOS）。类似地，74例表皮生长因子受体（epidermal growth factor receptor, *EGFR*）突变晚期非小细胞肺癌（non-small cell lung cancer, NSCLC）患者使用含双歧杆菌与乳杆菌的多菌株制剂后，PFS或OS亦无显著获益^[47,48]^。此外，部分研究^[49,50]^虽然显示PFS延长，但OS或ORR差异未达统计学意义。相比之下，丁酸梭菌MIYAIRI 588菌株（Clostridium butyricum MIYAIRI 588, CBM588）展现出获益证据的高度一致性。大规模双队列研究（*n*=927）及前瞻性研究^[51,52]^证实CBM588可延长PFS与OS。因此，推测不同菌株研究结果的巨大差异[如长双歧杆菌、鼠李糖乳杆菌GG削弱抗程序性细胞死亡配体1（programmed cell death ligand 1, PD-L1）疗效]，可能源于其免疫调节机制的迥异性^[53]^。此外，益生菌代谢产物或衍生物（如双歧杆菌来源的细胞外囊泡）亦可直接调控肿瘤微环境^[54]^。但益生菌对肺癌患者宿主基线菌群影响尚无明确结论。因此，应避免盲目使用非特异性益生菌，优先选择证据充分、机制明确的特定菌株；对近期接受广谱抗生素治疗者，可在ICIs治疗前引入具有菌群重建功能的益生菌以恢复微生态稳态，但需注意过度补充可能破坏菌群稳态^[55]^。相比之下，FMT通过整体重塑肠道菌群结构，可为肺癌精准治疗提供更系统、全面的新策略。

### 4.2 FMT治疗肺癌的作用机制

FMT的抗肿瘤作用核心在于通过多途径重塑肿瘤免疫微环境。首先，肠道菌群失调可引发过度细胞死亡，并释放营养物质促进致病菌（如沙门氏菌）过度增殖，加剧局部炎症并诱导化疗耐药^[56]^。而移植的有益菌群及其产物（如抗菌肽），可以诱导肺癌细胞发生线粒体介导的细胞凋亡，并将其细胞周期阻滞于S期，从而直接抑制肿瘤生长^[57]^。部分具有肿瘤靶向能力的细菌（如阿克曼氏菌）可在肺癌组织中定植^[58]^，并在动物模型中明确抑制肿瘤进展^[59]^。其次，FMT通过恢复有益的微生物代谢谱，尤其是SCFAs，发挥系统性免疫调节作用。SCFAs是FMT发挥疗效的重要媒介，具有维持肠道屏障、调节固有免疫与适应性免疫细胞的功能^[60,61]^。临床研究^[62]^证实，接受FMT治疗后，患者体内SCFAs（以丁酸盐为著）及产SCFAs菌群（如普拉梭菌）均显著提升，提示其作为疗效预测生物标志物的潜力；最后，FMT通过整体性重塑肠道菌群结构，逆转肺癌特征性的菌群失调，从而显著增强ICIs的疗效并减轻免疫相关不良反应^[63]^。重建的健康菌群能抑制促炎细胞因子（如IL-6、TNF-α）表达，并增强CD8^+^ T细胞等效应免疫细胞在肿瘤中的浸润，从而逆转免疫抑制微环境^[64]^。基于菌群与免疫的相互作用，通过FMT精准调控肠道生态，正逐步成为优化免疫治疗颇具前景的策略。

### 4.3 FMT治疗在肺癌领域的研究进展

FMT制备始于筛选出优质健康人群粪便，合格的粪便样本采集后保存于室温并于6 h内处理。将样本与约150 mL无菌氯化钠溶液混匀，过滤以清除大颗粒物，从而获得可用于移植的菌群悬液^[65]^。FMT已发展出多种给药途径，包括口服胶囊、鼻胃管/鼻十二指肠管和结肠镜（图2）。鉴于临床实际应用的需求，封装化FMT技术的研发也正迅速推进^[66]^。

**图 2:**
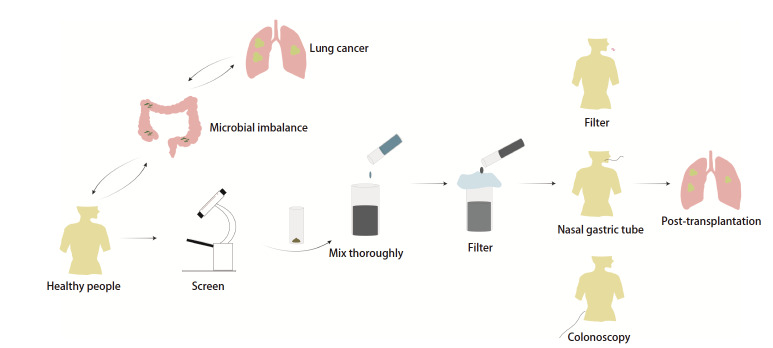
肺癌FMT治疗

#### 4.3.1 动物研究

近年来，动物研究已开始探索FMT在NSCLC治疗中的应用，并取得了颇具前景的结果。Ren等^[62]^收集了2021年11月至2023年8月接受铂类化疗联合ICIs治疗的41例NSCLC患者的粪便样本，移植至经抗生素处理的Lewis肺癌模型小鼠后，提升了ICIs的疗效，并伴随肿瘤增殖指数下降与宿主菌群多样性改善；Bi等^[67]^比较了不同来源菌群对肺癌进展的影响。移植阿尔茨海默病模型小鼠（代表一种疾病状态）的菌群，反而加速了肿瘤生长（体积增加约40%），其菌群以普雷沃菌属等条件致病菌富集为特征；相反，移植健康C57小鼠的菌群则显著抑制肿瘤（体积减少约47%），且伴随有益菌的增多与健康代谢功能的保留；Lee等^[68]^报道肺癌小鼠模型中，经过MS-20处理的小鼠粪便移植至受体小鼠，联合抗程序性细胞死亡受体-1（programmed cell death protein-1, PD-1）抗体治疗，抑制肿瘤生长。

#### 4.3.2 临床实践

首先，FMT在胃肠道疾病、黑色素瘤等领域的治疗应用已较为成熟，并展现出逆转免疫治疗耐药的潜力。Kim等^[69]^报告的临床试验（NCT04264975）中，13例PD-1抑制剂耐药的晚期实体瘤患者接受了供体来源FMT联合免疫治疗方案，其中6例出现持续性的菌群结构改善并实现临床获益，包括1例部分缓解和5例疾病稳定。另一项针对晚期黑色素瘤患者的I期研究^[70]^也观察到类似趋势，20例接受FMT治疗的患者中有13例有客观反应，提示FMT在增强免疫疗效方面具有跨癌种的适用潜力。其次，尽管FMT在肺癌领域的应用尚处于探索阶段，已有数据支持其安全性及初步有效性，这为未来肺癌治疗中开展精准微生态干预提供了新方向。Du等^[71]^报道，7例晚期NSCLC患者在接受健康供体来源的口服胶囊后再次接受免疫治疗，其中1例实现部分缓解，1例疾病稳定，PFS分别为14.6和8.1个月；Wei等^[72]^正在进行一项前瞻性临床试验，旨在评估FMT联合化疗-免疫方案一线治疗晚期NSCLC的疗效与安全性，其结果预计于2026年公布，有望为FMT在肺癌领域的规范应用提供关键依据。最后，随着技术发展，FMT正朝着更安全、标准化的方向升级。本文作者所在的南京医科大学第二附属医院张发明团队^[73]^开发的洗涤菌群移植技术（washed microbiota transplantation, WMT），相较于传统的FMT通过粪便重量的水量确定剂量和手动制备，WMT通过自动化系统去除粪便中的促炎代谢物、细菌碎片及潜在病原体，在保留功能菌群的同时显著提升移植安全性，并在清洗过程可定量富集菌群，实现精准干预。通过改善肠黏膜通透性和减少促炎代谢物前列腺素G2、白三烯B4、皮质酮产生，以降低FMT带来的如产超广谱β-内酰胺酶大肠杆菌菌血症等不良事件发生。与此同时，移植的菌群通过激活过氧化物酶体增殖物激活受体-γ（peroxisome proliferator-activated receptor-γ, PPAR-γ）信号通路促进Treg增殖^[74,75]^。在临床应用层面，针对酪氨酸激酶抑制剂（tyrosine kinase inhibitors, TKIs）引发的难治性胃肠道不良反应，WMT展现出确切疗效，其对腹泻、腹痛等症状的总体临床缓解率高达75.00%^[76]^，为基于微生态的个体化肿瘤支持治疗提供了循证依据。

## 5 总结与展望

FMT通过肠-肺轴调控宿主免疫，已成为增强肺癌免疫治疗、克服耐药及管理治疗相关毒性的一个极具潜力的策略。然而，作为一种成分复杂的生物制剂，其作用机制尚未完全解析。因此，未来研究需超越成分复杂的全菌群移植，转而聚焦于功能明确的工程化菌株组合。第一，鉴定并组装功能明确的下一代益生菌或合成菌群组合。这要求整合宏基因组学、代谢组学与免疫谱分析，在肺癌患者与健康对照中识别差异菌群及其功能通路，并通过人源化动物模型验证影响。第二，建立基于多组学数据的疗效预测模型，通过对FMT供体与受体进行高通量宏基因组测序等进行深度解析，提取关键微生物及代谢特征，并利用机器学习算法（如*LASSO*回归、随机森林）构建预测模型。经过严格验证后，利用可解释性人工智能（如SHAP值）揭示影响疗效的核心微生物决定因素。随着微生物组学、合成生物学与人工智能技术的交叉融合，FMT有望成为肿瘤多学科治疗标配。
